# Treatment of Cervicogenic Headache with Cervical Epidural Steroid Injection

**DOI:** 10.1007/s11916-014-0442-3

**Published:** 2014-08-05

**Authors:** Eugene Wang, Dajie Wang

**Affiliations:** Thomas Jefferson University Hospital, 834 Chestnut Street T150, Philadelphia, PA 19454 USA

**Keywords:** Cervicogenic headache, Cervical epidural steroid injections

## Abstract

Cervicogenic headache (CGH) is defined as referred pain from various cervical structures innervated by the upper three cervical spinal nerves. Such structures are potential pain generators, and include the atlanto-occipital joint, atlantoaxial joint, C2-3 zygapophysial joint, C2-3 intervertebral disc, cervical myofascial trigger points, as well as the cervical spinal nerves. Various interventional techniques, including cervical epidural steroid injection (CESI), have been proposed to treat this disorder. And while steroids administered by cervical epidural injection have been used in clinical practice to provide anti-inflammatory and analgesic effects that may alleviate pain in patients with CGH, the use of CESI in the diagnosis and treatment of CGH remains controversial. This article describes the neuroanatomy, neurophysiology, and classification of CGH as well as a review of the available literature describing CESI as treatment for this debilitating condition.

## Introduction

Cervicogenic headache (CGH) is a clinical syndrome characterized by primarily unilateral pain that originates in the neck, typically provoked by neck movement or pressure over tender points in the neck, with reduced range of movement of the cervical spine. Features of CGH headache can be similar to other primary headache disorders such as migraine, tension-type headache, and related cranial neuralgias such as occipital neuralgia. The diagnostic criteria for CGH headache put forth by the International Headache Society (IHS) are as follows [1]:

A. Pain, referred from a source in the neck and perceived in one or more regions of the head and/or face, fulfilling criteria C and D;
B. Clinical, laboratory, and/or imaging evidence of a disorder or lesion within the cervical spine or soft tissues of the neck known to be, or generally accepted as, a valid cause of headache;
C. Evidence that the pain can be attributed to the neck disorder or lesion based on at least one of the following:
  1. Demonstration of clinical signs that implicate a source of pain in the neck;
  2. Abolition of headache following diagnostic blockade of a cervical structure or its nerve supply using placebo- or other adequate controls;
D. Pain resolves within three months after successful treatment of the causative disorder or lesion.

### Various Pain Generators of CGH

CGH is thought to be referred pain arising from cervical structures innervated by the upper three cervical spinal nerves, and therefore any structure innervated by the C1–C3 spinal nerves could be the source for cervicogenic headache [2]. These nerves relay pain signals to the trigeminocervical nucleus, which is the nociceptive nucleus of the head and upper neck. This convergence is hypothesized to be responsible for referred pain to the occiput and/or eyes [3]. The potential pain generators of CGH are diverse; they include atlanto-occipital joint, atlantoaxial (AA) joints, C2-3 zygapophysial joint, C2-3 intervertebral disc, cervical myofascial trigger points, and the upper cervical spinal nerves themselves. Occipital neuralgia falls under a separate IHS heading, but may also produce CGH. The referred pain over the shoulder girdle, head, and upper arm can be either somatic referred pain (discogenic, facet joint-related, or myofascial) or radicular pain (nerve root irritation); pain in the forearm and hand is more likely to be radicular pain.

### Brief Review of Various Interventional Treatments for CGH

Different subtypes of CGH can be diagnosed and treated with various injections. For example, lateral atlantoaxial joint intra-articular injection can be effective for diagnosis and short-term pain relief for lateral atlantoaxial joint pain. This clinical entity is typically caused by osteoarthritis or post-traumatic event, and may account for up to 16 % of patients with CGH [4]. Symptoms include occipital or suboccipital pain, focal tenderness over the suboccipital area, restricted painful rotation of C1 on C2, and pain provocation by passive rotation of C1 [5]. Third occipital headache is secondary to C2-3 facet joint arthritis; this joint is innervated by the third occipital nerve, which is a superficial medial branch of C3 dorsal ramus [6]. This can be seen in 27 % of patients with CGH after whiplash injury from a motor vehicle accident. Tenderness over the C2-3 joint may be the only suggestive finding on physical examination [7]. Injection into this joint may produce pain relief, and radiofrequency ablation can be considered for long-term pain relief if temporary pain relief is achieved by third occipital nerve block [8]. Occipital neuralgia is coded separately under cranial neuralgias [1], but can produce symptoms indistinguishable from CGH. It typically presents as sharp pain in the occipital region, and this condition may require occipital nerve blockade for diagnosis and treatment. Nerve blocks to target the greater and lesser occipital nerves from dorsal ramus of C2 and C3 can be both diagnostic and therapeutic. Persistent headache secondary to occipital neuralgia may be amenable to occipital neurostimulation [9, 10].

C2 nerve root irritation or neuralgia is a subtype of occipital neuralgia that is caused by lesions affecting the C2 nerve root or dorsal ganglion such as neuroma, meningioma, or anomalous vessels. The C2 root lies posterior to the lateral atlantoaxial joint, and thus disorders or inflammation of this joint may lead to irritation or entrapment of the nerve root as well [11]. C2 neuralgia manifests as intermittent lancinating occipital pain, and may be associated with autonomic symptoms such as lacrimation, ciliary injection, and rhinorrhea. Abolition of pain by selective C2 nerve root block is essential for making an accurate diagnosis [5]. Cervical degenerative disease at C3-4, and particularly at C2-3, can produce CGH as well [1]. Cervical disc interventions for cervical discogenic pain are not commonly performed, however, due to the potential risk of esophageal penetration, leading to discitis or vascular injury [12]. Cervical myofascial pain from trigger points in the posterior neck muscles – particularly trapezius, sternocleidomastoid, and splenius capitis – has been proposed as a cause of headache [13, 14]. These tender points often overlie facet joints and may be indistinguishable from the latter as generators of pain. In addition, cervical myofascial pain frequently coexists with other acute and chronic painful musculoskeletal conditions, including CGH, cervical degenerative disc disease, cervical radiculopathy from nerve root irritation, cervical facet arthropathy, and chronic tension-type headache, although it may also be independent of other pain generators. A needling technique, with or without injectate, along with physical therapy, heat, and other conservative treatments, can provide pain relief. Certainly, all of the cervical spinal injections carry significant risks and should be performed by well-trained pain specialists. Cervical interlaminar epidural steroid injection (CESI) is one of the injections offered by pain specialists for treatment of CGH.

### Description and Evidence for Cervical Epidural Steroid Injection

Access to the cervical epidural space is available by both interlaminar and transforaminal approaches, and fluoroscopy is recommended for either approach [15–17]. In light of concerns regarding neurologic injury associated with transforaminal injection, interlaminar injections are still widely used, especially at the cervical level. One limitation with the interlaminar approach occurs in the case of obliteration of the posterior epidural space from previous surgery or focal central canal stenosis, which would make needle entry into the posterior epidural space difficult. Anatomical considerations for CESI are as follows:

The dimensions of the epidural space are smaller in the thoracic and cervical space compared with the lumbar region. The ligamentum flavum may not fuse in the midline, as this can be problematic when using the loss-of-resistance (LOR) technique. The epidural space is narrowed between C3 and C6 vertebral levels, about 2 mm on average [18]. The cervical interspaces with the largest interlaminar distance are typically found at C6/7 and C7/T1, which are the preferred entry points for most practitioners. Solutions injected into the epidural space preferentially flow along the dural sheaths of the spinal nerves, as the only significant barrier to epidural flow is the posterior longitudinal ligament. Thus fluid injected within the epidural space will tend to exit the spinal canal through the adjacent intervertebral foramina.

There is strong evidence that cervical/thoracic/lumbar interlaminar epidural steroid injection as a class of anesthetic treatment is effective in selected patients with radicular pain or radiculopathy. The duration of pain relief is variable. The recent retrospective controlled trials revealed evidence of short-term relief of radicular pain for up to six weeks [19]. Ferrante et al. attempted to find predictors of clinical outcome in a retrospective review of 100 patients who received CESI. In this study, radicular pain predicted a better outcome, and radiological diagnosis of a normal scan predicted a poor outcome, which suggests that appropriate candidates for CESI are patients with radicular pain correlating to physical and/or radiological findings [20•]. Strub et al. looked prospectively at the short-term benefits of CESI in 161 patients, and found that 83 % of injections resulted in pain relief. Patients with radicular symptoms into the fingers and with multilevel degenerative changes had a higher likelihood of success, while the odds of attaining pain relief were lower for patients requiring opiate analgesics [21], suggesting that epidural injections can be effective in the treatment of lumbar and cervical radicular pain. While cervical spinal nerve irritation is one of the etiologies of CGH, it is unclear whether CESI will be of benefit in the treatment of CGH. In this review, we evaluate the current evidence of CESI in the treatment of CGH.

## Methods

The literature referenced was obtained via a computer search of PubMed and MEDLINE from 1966 through March of 2014. The search terms included “cervicogenic headache,” “cervical epidural steroid injections,” “post-traumatic headache,” “treatment,” and “therapeutics.”

There have been no randomized controlled trials for treatment of CGH with CESI. Six articles were found according to the above search criteria. Two of these were review articles; three were small prospective case-control studies, of which one was a short letter to the editor to report longitudinal follow-up on a retrospective study. One was a retrospective observational study. Upon further inspection, we found that two of the prospective studies were by the same author and were virtually identical; therefore we were able to review only two studies on this topic.

## Results: Review of Available Studies for Treatment of CGH with CESI

Martelletti et al. conducted a prospective case-control study comparing Numerical Intensity Scale (NIS) and Drug Consumption Index (DCI) in nine patients with CGH (eight females, one male) and a control group of six sex- and age-matched patients (five females, one male) with chronic tension-type headache (CTH) [22••]. All CGH patients in the study had suffered from a continuous form of the disease for at least six months and were taking pain medications on a regular basis. The clinical diagnosis of CGH in these patients was confirmed with either greater occipital nerve or C2 nerve root blockade. Patients suffering from pain syndromes other than CGH were excluded. In consideration of the potential risk of the diagnostic technique, no placebo (i.e., saline solution) was used in the study. In order to examine unmasked conditions of both CGH and CTH, each patient underwent a 72-hour washout period from analgesics and anti-inflammatory medications when admitted as an inpatient. With the patients in sitting position and neck flexed, the C6-7 or C7-T1 interspinous space was palpated, and cutaneous infiltration anesthesia was administered with mepivacaine 1 % and sodium bicarbonate. In the midline approach, an 18 G Tuohy needle with syringe containing saline solution was inserted in the interspace, with loss-of resistance technique utilized to locate the epidural space; 40 mg of methylprednisolone mixed in 3–4 ml of saline was injected into the cervical epidural space. No radiographic guidance was mentioned in this paper.

A sharp decrease in NIS and DCI was observed in the CGH group treated with CESI compared with the CTH group. The short-term (12 hours) and medium-term (four weeks) clinical improvement in the CGH patients was statistically significant. The study results suggested that CESI could be beneficial in the treatment of CGH, as the authors hypothesized that this pain syndrome continued its course by “sensitizing the cervical nerve roots” and “initiating a pain-producing loop” involving nerve root and microvascular inflammation as well as mechanically-induced micro-injury. The authors also presented follow-up data on the longer-term effects of CESI on CGH. At the six-month endpoint comparing NIS and DCI values, the responses by CGH and CTH patients were not statistically different.

He et al. retrospectively analyzed 37 patients who underwent continuous CESI for CGH [23••]. The inclusion criteria in this observational study as follows: VAS score >60 mm during the pain period, radiographic evidence of C2-C6 disk degeneration and/or spondylosis, and positive response to blocking of C2 and C3 nerve roots prior to enrollment. The authors utilized either fluoroscopic or CT guidance for placement of the catheter in the cervical epidural space. As the authors approached the epidural space at the lower cervical/upper thoracic, they threaded a small catheter with steel wire 8-12 cm superior into the anterior epidural space. Lidocaine, dexamethasone, and saline were mixed together and infused at a rate of 5 mL/hour for three to four weeks. No control group was utilized in this study. In the first three months post-infusion, there was a significant decrease in number of days with mild to moderate pain, occurrence of severe pain, and NSAID usage. These parameters continued at improved rates in the next three months, but no significant differences were observed at 12 months. The results indicate that continuous epidural administration of local anesthetic and corticosteroid may control chronic CGH for at least six months. None of the patients reported any symptoms associated with corticosteroid-related adverse events such as gastroduodenal ulceration, hypothalamic-pituitary axis suppression, or infections.

## Conclusions

Accurate diagnosis of CGH is difficult due to the heterogeneity of its presentation and the multiple pain generators within the trigeminocervical nucleus-upper cervical nerve convergence. In addition, it is currently not possible to determine whether the pain generator is somatic referred or radicular-type pain, other than to attempt blockade of cervical nerve root, facet joint, atlantoaxial, myofascial trigger point, or occipital nerve steroid injection. Each cervical procedure carries potential risks. CESI with an interlaminar needle approach at C7-T1 or C6-7 epidural space is relatively safe compared to other cervical procedures. In the study by Martelletti et al., CESI was not proven to be of benefit in the long-term (six months) relief of CGH, but was helpful in the short term (up to four weeks). Based on prior studies of cervical epidural injections, it appears that radicular pain may respond to this treatment. One of the reasons that the Martelletti study failed to show a significant prolonged effect of CESI may be that the inclusion criteria of the study failed to selectively recruit patients with radicular pain from degenerative disc or spondylotic disease. Another consideration is that the authors used methylprednisolone alone, without a local anesthetic. The addition of a local anesthetic medication to the epidural steroid injection may prolong the effects of CESI. Finally, it is unclear whether fluoroscopic guidance, which has been shown to improve patient safety and injection accuracy, was utilized in the study [24, 25].

He et al.’s study of continuous epidural blockade for treatment of cervicogenic headache, although limited by lack of a control group, did show a decrease in the mean number of days patients experienced mild or moderate pain, the occurrence of severe pain, and daily NSAID usage at both three and six months. It is important to note that the inclusion criteria in this study comprised radiographic evidence of C2-C6 disk herniation, bulge, or degeneration, with positive response to blocking of C2 and C3 nerves for at least a one-week duration. This study revealed supporting evidence of CESI as an effective treatment for CGH when upper cervical disc pathologies are present. Cervical disc pathology is one of the etiologies for local nerve root irritation and injury. The injection of steroids in this location can inhibit aseptic inflammation and neurotransmission within the C-fibers. The continuous irritation of the most commonly involved nerve roots (C2, C3, C4) may produce inflammation and secondary swelling, which can perpetuate pain. Conceivably, injections specifically targeting these upper cervical nerve roots may be more effective. Interestingly, in He’s study, the epidural space was accessed at around the C7-T1 level. A steerable epidural catheter was placed through the needle and advanced 10 cm in the cephalad direction, which potentially targeted the upper cervical nerves responsible for persistent CGH. This method of injection to target the potential pain generators, along with the continuous infusion of corticosteroid/lidocaine over three to four weeks, may have contributed to the success of the study.

In both the He and Martelletti studies, no serious side effects were reported from epidural steroid injections. In the Martelletti study, one CGH patient and two CTH patients experienced a flushing sensation in the face, which lasted between 45 minutes and four hours post-injection. No long-term sequela of this symptom was seen. In the He study, none of the patients reported any symptoms associated with corticosteroid-related adverse events, such as hypothalamic-pituitary axis suppression, immunosuppression, or gastroduodenal ulceration. Based on the minimal adverse effects reported in these studies, it appears that CESI is a relatively safe injection therapy for CGH.

CGH is a complex medical condition that can be debilitating for patients and challenging for the treating physicians. The exact pathophysiology of this condition is not clear. Because of the heterogeneity of the presentation and the numerous pain generators that can cause CGH, it is often difficult to identify the source of this chronic pain condition. As a result, there are several different interventional techniques available for the diagnosis and treatment of CGH. CESI is considered by many interventional pain management specialists to be a reasonable option for patients who have failed conservative treatments. The two studies reviewed above provided preliminary evidence of both short- and long-term pain relief, particularly for patients with clinical and radiographic evidence of upper cervical spinal nerve root irritation. Further studies designed to include such patients with treatment protocols to target upper cervical nerve roots may provide additional evidence that CESI is an effective treatment for cervicogenic headache.
